# Faster fibrin clot degradation characterizes patients with central pulmonary embolism at a low risk of recurrent peripheral embolism

**DOI:** 10.1038/s41598-018-37114-4

**Published:** 2019-01-11

**Authors:** Robert W. Kupis, Sarah Goldman-Mazur, Maciej Polak, Michał Ząbczyk, Anetta Undas

**Affiliations:** 1 Krakow Centre for Medical Research and Technology, John Paul II Hospital, Krakow, Poland; 2 Department of Epidemiology and Population Studies, Institute of Public Health, Jagiellonian University Medical College, Krakow, Poland; 3 Institute of Cardiology, Jagiellonian University Medical College, Krakow, Poland

## Abstract

It is unclear whether thrombus location in pulmonary arteries is associated with particular clot characteristics. We assessed 156 patients following either central or peripheral pulmonary embolism (PE). Plasma clot lysis time, the rate of D-dimer release from plasma clots (D-D_rate_) with the maximum D-dimer concentration achieved (D-D_max_), as well as fibrin formation on turbidimetry, plasma clot permeation, thrombin generation, and fibrinolytic parameters were measured 3–6 months after PE. Patients following central PE (n = 108, 69.3%) were more likely smokers (38.9% vs 18.8%; p = 0.01), less likely carriers of factor XIII Val34Leu allele (40.7% vs 62.5%, p = 0.01), exhibited 16.7% higher D-D_rate_ and 12.7% higher tissue plasminogen activator antigen (tPA:Ag) compared with peripheral PE (p = 0.02 and p < 0.0001, respectively). Saddle PE patients (n = 31, 19.9%) had 11.1% higher D-D_rate_ and 7.3% higher D-D_max_ compared with central PE (both p < 0.05). Twenty-three recurrent PE episodes, including 15 central episodes, during a median follow-up of 52.5 months were recorded. Plasma D-dimer and tPA:Ag were independent predictors for central recurrent PE, whereas D-D_rate_ and peak thrombin predicted peripheral recurrent PE. Plasma clots degradation is faster in patients following central PE compared with peripheral PE and fibrinolysis markers might help to predict a type of recurrent PE.

## Introduction

Pulmonary embolism (PE) is a life-threatening disease which affects approximately 50 to 70 per 100 000 annually^ 1^ and causes more than 300 000 deaths in Europe per year^2^. PE can be classified either as central, when thrombus is located in the trunk or in the main pulmonary arteries, or peripheral, when it is seen in segmental or subsegmental arteries. Central arteries are most commonly involved^3^. The saddle PE, a thrombus straddling the bifurcation of the main pulmonary artery trunk, is found in 2–5% of all patients with PE^4^ and the short-term mortality in such patients is estimated at 4.3%, while the overall risk of death in all PE cases is estimated at 5.4%^5^. Factors that determine the location of embolic material in PE are largely unknown.

The last step of coagulation is the conversion of fibrinogen into insoluble fibrin, stabilized by active factor XIII. Fibrin properties determined by genetic and environmental factors^6,7^ including cigarette smoking or obesity^8^, can be unfavourably altered which in most cases denotes faster formation of compact fibre networks and relative resistance to plasmin. The prothrombotic clot phenotype has been shown in patients with a history of unprovoked venous thromboembolism (VTE), including those with PE and deep vein thrombosis (DVT)^9^. On the other hand, it has been demonstrated that clot lysis time (CLT) in acute PE is shorter, which correlates with lower clot density^10^, whereas residual pulmonary perfusion defects after acute PE were associated with longer CLT^11^. Recently, we have reported that the prothrombotic plasma clot phenotype, involving reduced permeation coefficient (K_s_) and reduced maximum rate of increase in D-dimer levels in the lysis assay (D-D_rate_), assessed a few months since the index event, is a risk factor for PE recurrences^12^.

It is unknown whether plasma clot properties differ with regard to the location of thrombotic material in pulmonary arteries. In central PE, it has been shown that the architecture of a surgically removed embolus depends on the location in pulmonary vasculature, with more tightly packed fibrin fibres in segmental pulmonary vessels^13,14^. The compaction of peripheral emboli may in part explain its resistance to endogenous fibrinolysis^15^. We hypothesized that plasma fibrin clots, which are degraded by fibrinolytic enzymes at a higher rate in specific assays, characterize patients with central PE. As a result, larger parts of thrombus could break off more easily, and might travel to the pulmonary arteries, where they would be lodged in larger calibre vessels.

## Material and Methods

### Patients

We investigated 156 consecutive patients aged 18–65 years with a history of documented first-ever PE episode referred to an outpatient clinic for thrombophilia screening or further laboratory work-up since June 2009 to March 2012. The inclusion and exclusion criteria were presented in detail previously^12^. Briefly, PE treated with thrombolysis or pulmonary embolectomy, known malignancy, end-stage renal insufficiency, liver injury, severe thrombophilia and ongoing anticoagulant treatment were the exclusion criteria. The diagnosis of DVT was based on positive colour duplex sonography findings. Based on the results of the high resolution spiral computed tomography, at least 64-row multidetector scanner, central PE was diagnosed when thrombi was located in the pulmonary trunk or/and in one of the main pulmonary arteries on the left or right side. Peripheral PE was diagnosed only when the thrombus was visualised in either segmental or subsegmental pulmonary arteries.

Vitamin K antagonists were started during the first week after the first-ever episode of PE, and continued for at least 3 months in cases of VTE that was triggered by short-term risk factors, or for at least 6 months in patients with VTE of unknown origin, based on the physicians’ decision.

The Jagiellonian University Ethical Committee approved the study and all patients gave the informed consent in accordance with the Declaration of Helsinki. All further methods and investigations were performed according to the newest and known guidelines and regulations.

### Laboratory investigations

Blood samples were drawn from an antecubital vein with minimal stasis using atraumatic and rapid venepuncture with at 8 to 10 AM following anticoagulation withdrawal. Citrate collection tubes were used. Lipid profiles, blood cell count, glucose, creatinine, and international normalized ratio (INR) were assayed by routine laboratory techniques. Fibrinogen was determined using the Clauss method. High-sensitivity C-reactive protein (CRP) was measured by nephelometry (Siemens, Marburg, Germany). Plasma D-dimer, tissue-type plasminogen activator (tPA:Ag) and plasminogen activator inhibitor-1 (PAI-1:Ag) antigens (all, American Diagnostica, Greewich, CT) were determined by immunoenzymatic assays. Factor V Leiden (FV Leiden), prothrombin 20210 A, FXIII Val34Leu and α-fibrinogen Thr312Ala polymorphisms were determined by the polymerase chain reaction followed by restriction fragment length polymorphism analysis, as previously described^9^. All measurements were performed by technicians blinded to the origin of the samples. Intra-assay and inter-assay coefficients of variation were 5–7%.

Fibrin clot analysis involving plasma clot permeability, turbidimetric assessment of clot formation and clot lysis at 2 various concentrations of recombinant tPA (rtPA) were performed in all PE patients as previously described^12^.

Briefly, the permeability of fibrin clot was determined using a pressure-driven system^9,16^. Tubes containing fibrin clots were connected through plastic tubing to a buffer reservoir, and the volume flowing through the gel was measured. The permeation coefficient (K_s_) was calculated from a formula:

$${{\rm{K}}}_{{\rm{s}}}=\mathrm{QxLx}\mu /\mathrm{txAx}{\rm{\Delta }}{\rm{p}},$$

where Q is the flow rate in time t; L, the length of a fibrin gel; µ, the viscosity of liquid (in poise); A, the cross-sectional area (in cm^2^) and Δp, a differential pressure (in dyne/cm^2^)^12^.

Plasma citrated samples were mixed 2:1 with a Tris buffer, containing 0.6 U/mL human thrombin (Sigma) and 50 mM calcium chloride, which initiated polymerization^9,17^. Absorbance was read at 405 nm. The lag phase of the turbidity curve, which reflects the time required for initial protofibril formation and maximum absorbance at the plateau phase (ΔAb_max_), indicating the number of protofibrils per fibre, were recorded^16,18^.

Efficiency of clot lysis was determined using 2 assays. In the first assay, CLT was measured as described^19,20^. Briefly, citrated plasma was mixed with 15 mM calcium chloride, 6 pM human tissue factor (Innovin, Siemens), 12 μM phospholipid vesicles and 14 µM recombinant tPA (rtPA, Boehringer Ingelheim, Germany). The mixture was transferred to a microtiter plate and its turbidity was measured at 405 nm at 37 °C. CLT was defined as the time from the midpoint of the clear-to-maximum-turbid transition, which represents clot formation, to the midpoint of the maximum-turbid-to-clear transition (representing the lysis of the clot). In the second assay, fibrin clots, formed as for the permeation evaluation were perfused with Tris buffer containing 0.2 µM rtPA and released D-dimer levels were measured in the effluent. D-dimer levels were measured every 30 minutes in the effluent. The experiment was stopped, usually after 90 to 120 min, while the fibrin gel collapsed under the pressure. D-D_rate_ and maximum D-dimer concentrations (D-D_max_) were analysed as described^9,16,21^.

Thrombin generation was assessed using the Calibrated Automated Thrombogram^22^. Briefly, 80 μl of thawed platelet poor plasma was mixed with 20 μl of a reagent containing recombinant relipidated tissue factor and phospholipids, with the final concentrations of 5 pM and 4 mM, respectively. Thrombin specific fluorogenic substrate was added. The fluorescence intensity was recorded by the Fluoroskan Ascent® microplate fluorometer (Thermo Fisher Scientific Oy, Vantaa, Finland) using the software program (Thrombinoscope BV, version 3.0.0.29). Three variables were analysed: “peak thrombin” (nM), i.e. the maximum concentration of thrombin formed during the registration time; the “endogenous thrombin potential” (ETP, nM × min), i.e. the area under the curve showing thrombin formation, and “time to thrombin peak” (s), i.e. the time from start of thrombin generation till the maximum thrombin value.

### Follow-up

We contacted all patients at least twice a year through clinic visits or by telephone. We used a confirmed symptomatic PE as the primary study endpoint. Secondary endpoints were all-cause death and DVT alone. Data were censored at the time of PE or DVT during follow-up. The spiral CT and pulmonary angiography were performed when recurrent PE was suspected. The colour duplex sonography was performed in every subject with signs or symptoms suggestive of DVT. VTE was considered as recurrent only if PE and/or DVT occurred after a successful acute treatment of the initial episode.

### Statistical analysis

The study was powered to have a 80% chance of detecting a 0.6 standardized mean difference in D-D_rate_ using a P-value of 0.05, based on the values from a published article^23^. In order to demonstrate such a difference or greater, 46 patients or more were required in each group. Data are expressed as mean and standard deviation (SD) or median (interquartile range, IQR) as appropriate. The Shapiro-Wilk test was used to assess conformity with a normal distribution. The continuous variables were compared between two groups using Student’s t test for independent groups for mean values and Mann-Whitney U test for distribution. Categorical variables were analysed using the χ^2^ test or Fisher’s exact test as appropriate. Predictors of central or peripheral recurrent PE episode were identified in the binominal logistic regression analysis (variables with p < 0.1 in the univariable analysis entered the respective multiple model). A strong correlation between any two parameters (r > 0.5) excluded one of the parameters from the multiple logistic regression models, except for fibrinogen, which was included as a key confounder. The final logistic regression model was adjusted for age, sex, BMI and fibrinogen. In the bivariate analysis all factors were adjusted for fibrinogen level separately. The Pearson’s correlation coefficient or Spearman’s rank correlation coefficient were calculated to assess correlations as appropriate. Two-sided P-values < 0.05 were considered statistically significant. Analysis was performed using SPSS 23.0 (SPSS Inc., Chicago, IL, USA).

## Results

A total of 156 patients were studied (Table 1). Initial central PE was observed in 108 cases (69.3%) including 31 patients with saddle PE (19.9%). Among 48 patients with peripheral PE (30.7%), 26 subjects had segmental PE (16.7%) and 22 patients had subsegmental PE (14.1%, Supplemental Table 1).

**Table 1:** Baseline characteristics of the studied groups.

Variable	Total (n = 156)	A Central PE (n = 108)	B Peripheral (n = 48)	p-value (A vs B)	Peripheral (n = 48)		p-value^#^ (A vs C vs D)
					C Segmental PE (n = 26)	D Subsegmental PE (n = 22)	
Age, years	44 ± 13	45 ± 12	41.08 ± 13.51	0.10	40 ± 15	43 ± 12	0.26
Male, n (%)	82 (52.6)	59 (54.6)	23 (47.9)	0.40	9 (34.6)	14 (64.0)	1.0
BMI, kg/m^2^	26.2 ± 4.3	25.89 ± 4.17	26.88 ± 4.53	0.29	27.07 ± 3.65	26.65 ± 5.46	0.31
Clinical characteristics							
Current Smoking, n (%)	51 (32.7)	42 (38.9)	9 (18.8)	0.01	7 (26.9)	2 (9.1)	0.02
Heart failure, n (%)	6 (3.8)	3 (2.8)	3 (6.3)	0.3	0 (0.0)	3 (14.0)	0.03
COPD, n (%)	6 (3.8)	5 (4.6)	1 (2.1)	0.4	0 (0.0)	1 (5.0)	0.54
Trauma/Surgery, n (%)	36 (23.1)	27 (25.0)	9 (18.8)	0.4	5 (19.0)	4 (18.0)	0.69
Pregnancy*, n (%)	10 (13.5)	5 (10.2)	5 (20.0)	0.2	3 (18.0)	2 (25.0)	0.37
Family history of VTE, n (%)	27 (17.3)	15 (13.9)	12 (25.0)	0.09	4 (15.0)	8 (36.0)	0.04
Oral Contraceptives*, n (%)	8 (12.2)	8 (10.8)	0 (0)	0.19	0 (0)	1 (20)	0.34
Unprovoked VTE, n (%)	89 (57.0)	60 (55.6)	29 (60.4)	0.6	16 (62.0)	13 (59.0)	0.84
Concomitant DVT, n (%)	53 (34.0)	33 (30.6)	20 (41.7)	0.2	11 (42.0)	9 (41.0)	0.40
Time of anticoagulation, months	10.6 ± 3.7	10.68 ± 3.67	10.42 ± 3.76	0.69	11.12 ± 4.37	9.59 ± 2.77	0.45
Laboratory parameters							
Creatinine, μmol/l	73.42 (12.4)	73.52 (13.4)	73.20 (9.9)	0.88	71.09 (10.5)	75.70 (8.6)	0.34
Glucose, mmol/l	5.04 (0.8)	4.98 (0.9)	5.0 (0.7)	0.77	5.05 (0.8)	5.00 (0.6)	0.70
TG, mmol/L	1.26 (0.8)	1.12 (0.7)	1.1 (0.8)	0.95	1.10 (1.0)	1.20 (0.7)	0.94
TC, mmol/l	5.09 (1.1)	5.16 (1.1)	4.92 (1.2)	0.21	4.93 (1.2)	3.04 (0.9)	0.36
HDL-C, mmol/l	1.49 (0.4)	1.48 (0.4)	1.49 (0.4)	0.92	1.50 (0.4)	4.90 (1.2)	0.87
LDL-C, mmol/l	3.14 (1.0)	3.21 (1.0)	3.00 (1.0)	0.24	2.98 (1.1)	1.48 (0.4)	0.26
hsCRP, mg/L	1.56 (1.6)	1.49 (1.3)	1.7 (1.0)	0.12	1.82 (1.3)	1.58 (0.7)	0.28
INR	0.98 (0.1)	0.98 (0.1)	0.97 (0.1)	0.66	0.98 (0.1)	0.97 (0.1)	0.91
D-dimer, ng/ml	295 (129)	297 (112)	293 (145)	0.82	302 (136)	283 (158)	0.65
Fibrinogen, g/l	3.1 (1.4)	2.98 (1.2)	3.5 (1.3)	0.03	3.41 (1.3)	3.70 (1.3)	0.09
Genetic polymorphisms, n (%)							
α-fibrinogen Thr312Ala allele carriers	52 (33.3)	34 (31.5)	18 (37.5)	0.5	6 (23.1)	12 (54.5)	0.054
Factor V Leiden mutation	14 (9.0)	12 (11.1)	2 (4.2)	0.16	0 (0.0)	2 (9.1)	0.21
Prothrombin 20210 A mutation	6 (3.8)	4 (3.70)	2 (4.2)	0.89	1 (3.8)	1 (4.5)	0.98
Factor XIII Val34Leu allele carriers	74 (47.4)	44 (40.7)	30 (62.5)	0.01	18 (69.5)	12 (54.5)	0.03

Data are shown as mean (standard deviation), median (interquartile range) or number (percentage). BMI, body mass index, COPD, chronic obstructive pulmonary disease; DVT, deep vein thrombosis; HDL-C, high-density lipoprotein cholesterol; hsCRP, high-sensitivity C-reactive protein; INR. international normalized ratio; LDL-C, low-density lipoprotein cholesterol; PE, pulmonary embolism; TC, total cholesterol; TG, triglycerides and VTE, venous thromboembolism. ^#^p- values refer to Kruskal-Wallis ANOVA test. *Females only.

Demographic and clinical parameters were similar in all groups, except for a higher prevalence of cigarette smokers in patients after central PE compared with the remainder (Table 1). In terms of routine laboratory investigations, fibrinogen was 15.6% lower in patients with central PE compared with peripheral PE (Table 1). No other intergroup differences in other laboratory parameters were observed.

Duration of anticoagulation treatment following the first episode of PE ranged from 4 to 20 months (median 10.5 months, Table 1). As shown in Table 1, factor XIII Val34Leu allele was found in 74 patients, and lower prevalence was found in the central PE group compared with those following subsegmental and subsegmental PE (p = 0.009 and p = 0.025, respectively). α-fibrinogen Thr312Ala, factor V Leiden and prothrombin 20210 A mutation were distributed similarly. No differences in genetic polymorphisms between patients with saddle PE and other types of central PE were observed (Supplemental Table 1).

### Fibrin variables

**Figure 1:**
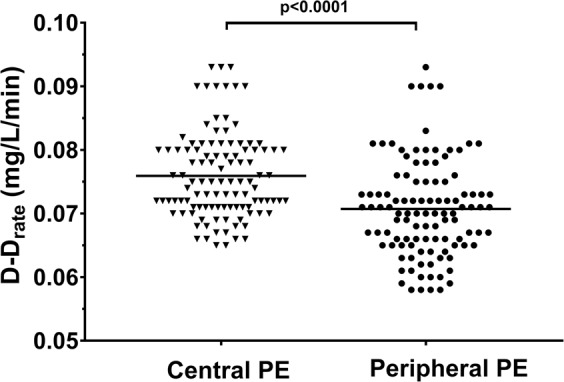
Comparison of maximum rate of increase in D-dimer levels in the lysis assay (D-D_rate_) between patients with central pulmonary embolism (PE) and peripheral PE.

Fibrin clot variables showed no differences among patients after the index central versus peripheral PE with the only exception. As shown in Fig. 1, after adjustment for fibrinogen, D-D_rate_ was 16.7% higher in the central PE group than in the peripheral PE group, including individuals with segmental and subsegmental episodes (by 15.4% and by 13.6%, respectively).

**Figure 2:**
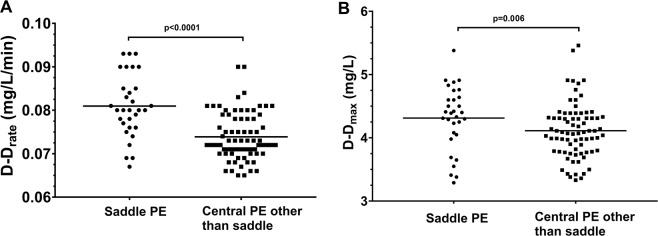
Comparison of maximum rate of increase in D-dimer levels in the lysis assay (D-D_rate_, panel A) and maximum D-dimer levels in the lysis assay (D-D_max_, panel B) between group with saddle pulmonary embolism (PE) vs group with other central PE.

When patients with saddle PE were compared with other subjects with central PE, we found that, after adjustment for fibrinogen, D-D_rate_ was 11.1% higher and D-D_max_ 7.3% higher compared with the latter group (Fig. 2, panel A and B; Supplemental Table 1). No other intergroup differences in fibrin clot properties were observed.

Moreover, tPA:Ag was higher in patients with central PE compared with patients with peripheral (p = 0.006) and both segmental and subsegmental PE (by 12.9% and by 10.6%, p = 0.02; Table 2). Saddle PE patients showed 16.7% higher tPA:Ag than other central PE (Supplemental Table 2). In the central PE group D-D_rate_ correlated positively with D-D_max_ (r = 0.2, p = 0.03) and tPA:Ag (r = 0.28, p = 0.003), whereas in the peripheral PE D-D_rate_ was positively associated solely with tPA:Ag (r = 0.23, p = 0.003). There were no intergroup differences in PAI-1:Ag.

**Table 2:** Comparison of fibrin clot features, thrombotic and fibrinolysis markers in the studied groups.

Variable	Total (n = 156)	A Central PE (n = 108)	B Peripheral (n = 48)	p-value (A vs B)	Peripheral (n = 48)		p-value (A vs C vs D)
					C Segmental PE (n = 26)	D Subsegmental PE (n = 22)	
Lag phase, s	41.84 (5.1)	41.69 (5.1)	42.19 (5.3)	0.57	42.38 (5.6)	41.95 (5.2)	0.76
ΔAb_max_, 405 nm	0.83 (0.1)	0.82 (0.1)	0.84 (0.1)	0.12	0.85 (0.1)	0.84 (0.1)	0.23
K_s_, 10^−9^ cm^2^	7.24 (1.1)	7.32 (1.1)	7.07 (1.2)	0.19	7.07 (1.2)	7.07 (1.2)	0.42
D-D_max_, mg/L	4.14 (0.5)	4.17 (0.5)	4.07 (0.6)	0.24	4.04 (0.6)	4.09 (0.6)	0.30
D-D_rate_, mg/L/min^ǂ^	0.072 (0.011)	0.075 (0.009)	0.065 (0.0065)	<0.0001	0.065 (0.005)	0.066 (0.008)	<0.0001
CLT, min	87.89 (16.2)	87.94 (16.9)	87.8 (14.7)	0.95	89.23 (14.2)	86.05 (15.4)	0.74
Time to peak, s	310.5 (142.5)	307.00 (132.0)	315.50 (150.5)	0.39	326.50 (119.0)	285.00 (187.0)	0.32
Peak thromin, nM	240.41 (84.2)	241.00 (83.8)	239.50 (90.8)	0.67	237.00 (79.0)	269.50 (132.6)	0.40
ETP, nM × min	1578.3 (92.7)	1576.6 (93.6)	1582.3 (91.3)	0.72	1564.19 (89.7)	1603.64 (90.5)	0.40
tPA:Ag, ng/mL	9.83 (2.8)	10.21 (2.7)	9.0 (2.9)	0.006	8.89 (2.8)	9.13 (3.0)	0.02
PAI-1:Ag, ng/ml^ǂ^	12.9 (7.34)	12.90 (7.25)	12.0 (7.22)	0.56	12.10 (6.7)	11.50 (6.5)	0.69

Data are shown as median (interquartile range). ΔAb_max_, maximum absorbance on turbidimetry; CLT, clot lysis time; D-D_max_, maximum D-dimer levels in the lysis assay; D-D_rate_, maximum rate of increase in D-dimer levels in the lysis assay; ETP, endogenous thrombin potential; K_s_, fibrin clot permeability coefficient; PAI-1:Ag, plasminogen activator inhibitor-1 antigen; peak thrombin, peak thrombin concentration; time to peak, time to peak thrombin generation; tPA:Ag, tissue plasminogen activator antigen. ^ǂ^Adjusted for fibrinogen.

There were no differences in thrombin generation profiles, including time to peak, peak thrombin and ETP, between central and peripheral PE. The same held true for individuals with saddle PF versus other central PE (Table 2, Supplemental Table 2).

Smokers exhibited 3.6% lower ΔAb_max_ than non-smokers, while K_s_ was 4% higher in smokers than non-smokers (Supplemental Table 3). No other differences in fibrin clot variables between the smoking and non-smoking groups were observed.

Patients with FXIII Val34Leu allele exhibited lower K_s_ (median, [IQR]; 6.0 [1.6] 10^−9^ cm^2^ vs 7.45 [1.3] 10^−9^ cm^2^, respectively, p = 0.016) and higher ΔAb_max_ (median, [IQR]; 0.85 [0.11] 405 nm vs 0.81 [0.8] 450 nm, respectively, p = 0.005) compared with the non-carriers. Moreover, tPA:Ag was lower in the 34 Leu carriers group (median, [IQR]; 9.1 [4.0] ng/mL vs 10.2 [3.6] ng/mL, respectively, p = 0.008). No other intergroup differences related to genetic polymorphisms were observed.

#### Follow-Up

The median follow-up was 52.5 months (range 2 to 70 months). Five (3.2%) patients were lost to follow-up. Twenty-three (14.7%) recurrent PE episodes were recorded. In 15 (65.2%) patients central recurrent PE episode was reported, including 7 (30.4%) saddle PE episodes). There were 5 (21.7%) segmental and 3 (13.0%) subsegmental recurrent PE episodes. Median time from the first PE episode to the recurrent PE episode was 16 months (IQR, 14 months). Seven (4.5%) patients died, including 2 patients who experienced recurrent PE (one central and one segmental).

No differences in demographic and laboratory parameters were observed in patients with recurrent central versus peripheral PE (Supplemental Table 4). No differences were shown in regard to fibrin clot features, thrombotic and fibrinolysis markers compared peripheral with no-saddle central recurrent PE, including no differences in either D-D_rate_ or D-D_max_ (Supplemental Table 5).

Multinominal multivariate logistic regression adjusted for sex, age, BMI and fibrinogen showed that plasma D-dimer and tPA:Ag were predictors for central recurrent PE, whereas D-D_rate_ and peak thrombin predicted peripheral recurrent PE (Table 3). Every 0.01 mg/L/min increase in D-D_rate_ was associated with lower risk of peripheral PE (OR 0.09; 95% CI, 0.01–0.66; p = 0.02), whereas every 100 ng/ml increase in plasma D-dimer was predictive of central recurrent PE (OR, 1.006; 95% CI, 1.002–1.010; p = 0.001; Table 3). Similar results were observed in bivariate multinominal regression built independently for each factor with adjustment for fibrinogen (Supplemental Table 6).

**Table 3:** Multinominal logistic regression model for central or peripheral recurrent PE episode in relation to recurrence-free patients.

Variable	Central PE recurrence				Peripheral PE recurrence			
	Univariate analysis		Multivariate analysis		Univariate analysis		Multivariate analysis	
	OR (95% CI)	p-value	OR (95% CI)	p-value	OR (95% CI)	p-value	OR (95% CI)	p-value
Age	1.02 (0.97–1.06)	0.48	1.02 (0.97–1.07)	0.53	1.03 (0.97–1.09)	0.40	1.04 (0.97–1.12)	0.28
Male sex	1.88 (0.63–5.59)	0.26	3.22 (0.79–13.14)	0.10	3.76 (0.73–19.33)	0.11	5.97 (0.69–51.60)	0.10
BMI	0.99 (0.87–1.12)	0.87	0.93 (0.78–1.12)	0.46	1.02 (0.86–1.20)	0.84	0.93 (0.75–1.17)	0.54
Fibrinogen	0.47 (0.22–0.99)	0.047	0.27 (0.09–0.78)	0.016	0.93 (0.42–2.06)	0.86	0.51 (0.16–1.59)	0.24
D-dimer	1.005 (1.002–1.008)	0.002	1.006 (1.002–1.010)	0.001	1.003 (0.999–1.007)	0.1	1.002 (0.997–1.006)	0.44
K_s_	0.92 (0.56–1.50)	0.7	—	—	0.54 (0.26–1.10)	0.09	—	—
D-D_max_	0.84 (0.29–2.44)	0.74	—	—	0.84 (0.20–3.52)	0.81	—	—
D-D_rate_	0.55 (0.26–1.18)	0.12	0.57 (0.19–1.67)	0.30	0.21 (0.06–0.74)	0.02	0.09 (0.01–0.66)	0.02
peak thrombin	1.003 (0.996–1.011)	0.4	1.003 (0.993–1.014)	0.55	1.012 (1.004–1.020)	0.003	1.016 (1.005–1.027)	0.004
ETP	1.001 (0.995–1.007)	0.8	—	—	1.008 (0.999–1.017)	0.07	—	—
tPA:Ag	0.78 (0.62–0.98)	0.03	0.70 (0.51–0.96)	0.03	0.96 (0.74–1.25)	0.78	0.77 (0.52–1.15)	0.20

The final model was adjusted for: age, sex, BMI and fibrinogen. BMI, body mass index; ETP, endogenous thrombin potential; D-D_max_, maximum D-dimer levels in the lysis assay; D-D_rate_, maximum rate of increase in D-dimer levels in the lysis assay; ETP, endogenous thrombin potential; K_s_, fibrin clot permeability coefficient; peak thrombin, peak thrombin concentration; tPA:Ag, tissue plasminogen activator antigen.

## Discussion

To our knowledge, this study is the first to evaluate associations between plasma fibrin clot properties and the PE location with long-term follow-up. We demonstrated that patients with central PE display higher rate of fibrin clot degradation, reflected by increased D-D_rate_, compared with peripheral PE. The difference in the rate of fibrinolysis was even more pronounced, when saddle PE patients were compared with those with other central PE episodes. Moreover, higher D-D_rate_ is associated with lower risk of recurrent peripheral PE. This study suggests that there is a persistent tendency to faster fragmentation of solid preformed fibrin, measurable in a plasma-based assay 3–6 months after the index PE event that can be observed in subjects who experienced central PE. Our study suggests that there are some individual differences in clot lysability that may distinguish different PE forms and some phenotypic characteristics of fibrinolysis might determine the size of degraded thrombi visualized on angio-CT.

From a methodological point of view, we found that a single lysis assay is able to differentiate between patients with central or peripheral PE when tested after the event. This assay determines the rate of rtPA-induced degradation of the previously prepared plasma clots (using thrombin and calcium added to citrated plasma) by evaluating D-D_rate_. However, differences in fibrinolysis between those two subgroups were absent while using CLT in which a much lower rtPA concentration is applied together with phospholipids and tissue factor. This discrepancy between results of those two assays has been observed previously in a few disease states^23,24^. It might be speculated that fibrinolysis as a complex closely regulated process cannot be assessed for clinical purposes in different conditions using one assay. One assay, like one size, does not fit all. Interestingly, in the context of PE, the assay describing the degradation of fibrin clot at higher rtPA concentrations in the buffer allows to detect individuals prone to develop central PE, in particular the saddle PE. Of note, the assay in which D-D_rate_ or D-D_max_ are evaluated, resembles the situation, in which already existing clots in veins and/or pulmonary arteries are exposed to exogenous tPA during fibrinolytic therapy. The concentrations of rtPA used in this assay are similar to those encountered during thrombolysis^25^.

In our study a larger prevalence of smokers in patients following central PE is an intriguing finding. Smoking has been reported to be an independent risk factor of reduced long-term survival in VTE patients^26^. To our knowledge, no differences in PE location in regard to smoking have been reported in the literature. It is worth noting that current smoking may unfavourably alter clot properties with the greatest impact observed in heavy smokers^27^. Moreover, smoking cessation results in improved fibrin clot properties, reflected by less dense fibrin network with wider branching angles between fibres^27^.

A higher prevalence of FXIII Val34Leu allele carriers among subjects with peripheral PE deserves a comment. FXIII 34Leu allele has been reported to be associated with lower clot permeation and turbidity compared to 34Val^28^, which is consistent with our study. A protective role of 34Leu in thromboembolism is supported by few studies^29^. The unexpected association between PE location and these polymorphisms is a novel finding with deserves further investigation.

We observed that higher D-D_rate_ is associated with lower risk of recurrent peripheral PE. Previous studies done by our group have shown that prothrombotic clot phenotype is associated with higher risk of recurrent VTE and PE events^12,30^. The decision how long the treatment should last in PE patients is made in most cases in a individual manner taking into account patient risks and benefits. Usually, the treatment lasts 3–6 months, however, for unprovoked PE extended time of treatment seems to be beneficial^31^. Our finding suggest that patients with unfavourable clot properties after provoked or unprovoked PE should be under closer surveillance if they discontinue anticoagulation. It is possible that patients with prothrombotic clot phenotype could benefit from longer anticoagulation to prevent PE recurrences. Given intensive efforts to standardize fibrin clot measurements, we believe that the diagnostic potential of these new markers is substantial, however, further work on implementation of such measurements in practice are needed.

This study has several limitations. Firstly, the size of the recurrent PE group was small, but similar to values reported in other studies^11^. It is unknown whether the results obtained 3–6 months after the event could have been observed during acute PE, because there was no data about fibrin clot properties measured at the time of diagnosis. We cannot extrapolate our findings to the high-risk or elderly PE patients who were ineligible in this study. Secondly, blood samples were drawn only once; therefore, we cannot exclude changes in fibrin clot properties over time. Plasminogen or plasma factor XIII levels were not measured. Oligo- or asymptomatic recurrent PE or DVT could have been overlooked and the PE recurrences might have been underrepresented. Finally, since clot properties are affected by a number of factors, other potential modulators of plasma fibrin clot structure and function following the first PE episode might also be important, and they should be investigated in the future.

In conclusion, our study showed that the rate of clot degradation at concentrations of rtPA similar to those during thrombolysis is enhanced in patients with central PE and decreases when the pulmonary artery calibre reduces. D-D_rate_ might help to predict form of recurrent PE episodes.

## Supplementary information

Supplemental Tables
