# Supplementary material for: Faster fibrin clot degradation characterizes patients with central pulmonary embolism at a low risk of recurrent peripheral embolism
Source: Sci Rep. 2019 Jan 11;9:72. doi: 10.1038/s41598-018-37114-4 (PMC6329786; doi:10.1038/s41598-018-37114-4)
Supplement: Supplementary file 1 — Supplemental Tables [file 41598_2018_37114_MOESM1_ESM.docx]

**Faster fibrin clot degradation characterizes patients with central pulmonary embolism at a low risk of recurrent peripheral embolism.**

Robert W. Kupis^1,*^, Sarah Goldman-Mazur^1,*^, Maciej Polak^2^, Michał Ząbczyk^1^, Anetta Undas^1,3^

^1^Krakow Centre for Medical Research and Technology, John Paul II Hospital, Krakow;

^2^ Department of Epidemiology and Population Studies, Institute of Public Health, Jagiellonian University Medical College, Krakow, Poland;

^3^Institute of Cardiology, Jagiellonian University Medical College, Krakow, Poland

*equally contributed

Corresponding author:

Anetta Undas, MD, PhD

Institute of Cardiology, Jagiellonian University Medical College

80 Prądnicka Str.

31-202 Kraków, Poland

Phone no: +48126143004

Fax no: +48126142021

Email address: mmundas@cyf-kr.edu.pl

Supplemental Table 1. Characteristics of patients with saddle PE vs central PE other than saddle.

| Variable | Total central PE,  n=108 | A  Saddle PE  n=31 | B  Other central PE  n=77 | p-value  (A vs B) |
| --- | --- | --- | --- | --- |
| Age, years | 45±12 | 45 (15) | 46 (20) | 0.99 |
| Male, n (%) | 59 (54.6) | 17 (54.8) | 42 (54.5) | 0.98 |
| BMI, kg/m2 | 25.89±4.17 | 24.1 (5.6) | 26.1 (6.0) | 0.10 |
| Clinical characteristics | | | | |
| Smoking, n (%) | 42 (38.9) | 9 (29) | 33 (42.9) | 0.18 |
| Heart failure, n (%) | 3 (2.8) | 0 (0) | 3 (3.9) | 0.27 |
| COPD, n (%) | 5 (4.6) | 1 (3.2) | 4 (5.2) | 0.66 |
| Trauma/Surgery, n (%) | 27 (25.0) | 9 (29) | 18 (23.4) | 0.54 |
| Pregnancy*, n (%) | 5 (10.2) | 2 (14.3) | 3 (8.6) | 0.57 |
| Family history of VTE, n (%) | 15 (13.9) | 4 (12.9) | 11 (14.3) | 0.85 |
| Contraceptives*, n (%) | 8 (16.3) | 3 (21.4) | 5 (6.5) | 0.57 |
| Idiopathic VTE, n (%) | 60 (55.6) | 17 (54.8) | 43 (55.8) | 0.92 |
| PE and DVT, n (%) | 33 (30.6) | 7 (22.6) | 26 (33.8) | 0.25 |
| Time of anticoagulation, months | 10.68±3.67 | 10 (4) | 11 (4) | 0.71 |
| Laboratory parameters | | | | |
| Creatinine, μmol/l | 73.52(13.4) | 70.72 (17.68) | 70.72 (20.0) | 0.91 |
| Glucose, mmol/l | 4.98 (0.9) | 4.95 (0.83) | 5.0 (0.95) | 0.40 |
| TG, mmol/L | 1.12 (0.7) | 1.18 (0.43) | 1.1 (0.84) | 0.36 |
| TC, mmol/l | 5.16 (1.1) | 5.47 (1.95) | 5.13 (1.48) | 0.47 |
| HDL-C, mmol/l | 1.48 (0.4) | 1.45 (0.64) | 1.42 (1.5) | 0.76 |
| LDL-C, mmol/l | 3.21 (1.0) | 3.32 ± 0.97 | 3.16 ± 1.00 | 0.88 |
| hsCRP, mg/L | 1.49 (1.3) | 1.75 (1.40) | 1.35 (1.28) | 0.37 |
| INR | 0.98 (0.1) | 0.997 ± 0.09 | 0.971 ± 0.09 | 1.0 |
| D-dimer, ng/ml | 296.50 (111.5) | 286 (120) | 299 (119) | 0.12 |
| Fibrinogen, g/l | 2.98 (1.2) | 2.99 (1.85) | 2.97 (1.04) | 0.69 |
| Genetic polimorphisms, n (%) | | | | |
| α-fibrinogen Thr312Ala allele carriers | 34 (31.5) | 7 (22.6) | 27 (35.1) | 0.21 |
| Factor V Leiden mutation | 12 (11.1) | 5 (16.1) | 7 (9.1) | 0.29 |
| Prothrombin 20210A mutation | 4 (3.70) | 1 (3.2) | 3 (3.9) | 0.87 |
| Factor XIII Val34Leu allele carriers | 44 (40.7) | 11 (35.5) | 33 (42.9) | 0.48 |

Data are shown as mean (standard deviation), median (interquartile range) or number (percentage). BMI, body mass index, COPD, chronic obstructive pulmonary disease; DVT, deep vein thrombosis; HDL-C, high-density lipoprotein cholesterol; hsCRP, high-sensitivity C-reactive protein; INR. international normalized ratio; LDL-C, low-density lipoprotein cholesterol; PE, pulmonary embolism; TC, total cholesterol; TG, triglycerides and VTE, venous thromboembolism. *Females only

Supplemental Table 2. Comparison of fibrin clot features, thrombotic and fibrinolysis markers between patients with saddle PE and other central PE.

| Variable | Total central PE,  n=108 | Saddle PE,  n=31 | Other central PE,  n=77 | p-value |
| --- | --- | --- | --- | --- |
| Lag phase, s | 41.69 (5.1) | 41.2 ±5.1 | 41.9 ± 5.1 | 1.0 |
| ΔAb_max_, 405 nm | 0.82 (0.1) | 0.83 (0.09) | 0.82 (0.09) | 0.66 |
| K_s_, 10^–9^ cm^2^ | 7.32 (1.1) | 7.18±0.83 | 7.37 ±1.14 | 0.053 |
| D-D_max_, mg/L | 4.17 (0.5) | 4.39 (0.59) | 4.09 (0.53) | 0.017 |
| D-D_rate_, mg/L/min^ǂ^ | 0.075 (0.009) | 0.080 (0.009) | 0.072 (0.007) | <0.0001 |
| CLT, min | 87.94 (16.9) | 88.0 (23.0) | 90.0 (28.0) | 0.83 |
| Time to peak, s | 307.00 (132.0) | 289 (97) | 317 (137) | 0.054 |
| Peak thromin, nM | 241.00 (83.8) | 240.0 (85.9) | 243.1 (78.9) | 0.25 |
| ETP, nM×min | 1576.6 (93.6) | 1567.2 ± 98.4 | 1580.3 ±92.0 | 0.63 |
| tPA:Ag, ng/mL^ǂ^ | 10.21 (2.7) | 11.2 (2.8) | 9.6 (3.0) | 0.006 |
| PAI-1:Ag, ng/ml | 12.90 (7.25) | 13.5 (5.9) | 12.9 (7.4) | 0.13 |

Data are shown as mean (standard deviation) or median (interquartile range). ΔAb_max_, maximum absorbance on turbidimetry; CLT, clot lysis time; D-D_max_, maximum D-dimer levels in the lysis assay; D-D_rate_, maximum rate of increase in D-dimer levels in the lysis assay; ETP, endogenous thrombin potential; K_s_, fibrin clot permeability coefficient; PAI-1:Ag, plasminogen activator inhibitor-1 antigen; peak thrombin, peak thrombin concentration; time to peak, time to peak thrombin generation; tPA:Ag, tissue plasminogen activator antigen. ^ǂ^ adjusted for fibrinogen.

Supplemental Table 3. Comparison of fibrin clot features, thrombotic and fibrinolysis markers between smokers and non-smokers

| Variable | Smokers, n=51 | Non-smokers, n=105 | p-value |
| --- | --- | --- | --- |
| Lag phase, s | 42.25 (9.0) | 41.0 (7) | 0.31 |
| ΔAb_max_, 405 nm | 0.81 (0.1) | 0.84 (0.1) | 0.045 |
| K_s_, 10^–9^ cm^2^ | 7.46 (1.1) | 7.14 (1.1) | 0.03 |
| D-D_max_, mg/L | 4.03 (0.5) | 4.19 (0.5) | 0.07 |
| D-D_rate_, mg/L/min^ǂ^ | 0.07 (0.01) | 0.07 (0.01) | 0.35 |
| CLT, min | 86.29 (26.0) | 90.0 (24.0) | 0.51 |
| Time to peak, s | 332.12 (138.0) | 299.0 (143.0) | 0.41 |
| Peak thrombin, nM | 252.09 (83.43) | 241.57 (84.8) | 0.62 |
| ETP, nM×min | 1576.63 (117.0) | 1588.0 (116.0) | 0.72 |
| tPA:Ag, ng/mL | 9.63 (3.3) | 10.2 (3.8) | 0.39 |
| PAI-1:Ag, ng/ml | 14.3 (8.3) | 12.9 (6.9) | 0.41 |

Data are shown as mean (standard deviation) or median (interquartile range). Abbreviations: see Supplemental Table 2.

Supplemental Table 4. Characteristics of patients with central vs peripheral episode of recurrent PE.

| Variable | Total recurrent PE  n=23 | A  Central recurrent PE  n=15 | B  Peripheral recurrent PE  n=8 | p-value  (A vs B) |
| --- | --- | --- | --- | --- |
| Age, years | 46 (19) | 46 (19) | 50 (16.5) | 0.87 |
| Male, n (%) | 8 (34.8) | 6 (42.9) | 0 (0) | 0.47 |
| BMI, kg/m2 | 25.7 (5) | 25.4 (5) | 26.3 (4.2) | 0.65 |
| Clinical characteristics | | | | |
| Smoking, n (%) | 5 (21.7) | 5 (35.7) | 2 (22.2) | 0.06 |
| Heart failure, n (%) | 2 (6.7) | 2 (14.3) | 0 (0) | 0.28 |
| COPD, n (%) | 1 (4.4) | 1 (7.1) | 0 (0) | 0.46 |
| Trauma/Surgery, n (%) | 1 (4.4) | 1 (7.1) | 0 (0) | 0.46 |
| Pregnancy*, n (%) | 3 (20) | 3 (27.3) | 0 (0) | 0.18 |
| Family history of VTE, n (%) | 4 (17.4) | 1 (7.1) | 3 (33.3) | 0.06 |
| Contraceptives*, n (%) | 0 (0) | 0 (0) | 0 (0) | - |
| Idiopathic VTE, n (%) | 17 (73.9) | 10 (71.4) | 7 (77.8) | 0.28 |
| PE and DVT, n (%) | 11 (47.8) | 7 (50) | 4 (44.4) | 0.88 |
| Time of anticoagulation, months | 12 (4) | 12 (4) | 13 (2) | 0.29 |
| Laboratory parameters | | | | |
| Creatinine, μmol/l | 71.0 (20.0) | 75.0 (21.0) | 70.5 (11.0) | 0.29 |
| Glucose, mmol/l | 5.1 (3.1) | 5.2 (1.4) | 5.0 (0.6) | 0.30 |
| TG, mmol/L | 1.14 (0.95) | 1.13 (1.12) | 1.19 (0.37) | 0.90 |
| TC, mmol/l | 4.84 (1.98) | 4.77 (1.63) | 5.03 (2.08) | 0.97 |
| HDL-C, mmol/l | 1.52 (0.51) | 1.53 (0.56) | 1.43 (0.40) | 0.61 |
| LDL-C, mmol/l | 3.0 (1.5) | 3.0 (1.88) | 3.1 (1.48) | 0.77 |
| hsCRP, mg/L | 1.82 (2.75) | 1.57 (3.08) | 2.13 (2.27) | 0.44 |
| INR | 0.980 (0.10) | 0.980 (0.10) | 0.975 (0.14) | 0.70 |
| D-dimer, ng/ml | 390 (256) | 369 (330) | 422 (212) | 0.65 |
| Fibrinogen, g/l | 2.9 (1.13) | 2.7 (0.73) | 3.2 (1.18) | 0.25 |
| Genetic polimorphisms, n (%) | | | | |
| α-fibrinogen Thr312Ala allele carriers | 10 (43.5) | 6 (42.9) | 4 (44.4) | 0.65 |
| Factor V Leiden mutation | 1 (4.4) | 0 (0) | 1 (11.1) | 0.16 |
| Prothrombin 20210A mutation | 1 (4.4) | 1 (7.1) | 0 (0) | 0.46 |
| Factor XIII Val34Leu allele carriers | 14 (60.9) | 9 (64.3) | 5 (55.6) | 0.91 |

Data are shown as mean (standard deviation), median (interquartile range) or number (percentage). Abbreviations: see Supplemental Table 1. *Females only

Supplemental Table 5. Comparison of fibrin clot features, thrombotic and fibrinolysis markers between patients with central and peripheral recurrent PE episode.

| Variable | Total recurrent PE,  n=23 | Central recurrent PE,  n=15 | Peripheral recurrent PE,  n=8 | p-value |
| --- | --- | --- | --- | --- |
| Lag phase, s | 40 (7) | 40 (7) | 40 (9) | 0.67 |
| ΔAb_max_, 405 nm | 0.83 (0.12) | 0.80 (0.11) | 0.87 (0.12) | 0.46 |
| K_s_, 10^–9^ cm^2^ | 6.9 (1.7) | 6.9 (1.0) | 6.1 (2.5) | 0.22 |
| D-D_max_, mg/L | 4.1 (0.6) | 4.0 (0.5) | 4.2 (0.8) | 0.87 |
| D-D_rate_, mg/L/min | 0.070 (0.007) | 0.071 (0.006) | 0.067 (0.009) | 0.14 |
| CLT, min | 92 (16) | 92 (30) | 92.5 (12.5) | 0.58 |
| Time to peak, s | 329 (180) | 310 (180) | 368 (152) | 0.58 |
| Peak thromin, nM | 286.5 (74.0) | 275.0 (102.0) | 306.4 (127.0) | 0.08 |
| ETP, nM×min | 1585 (140) | 1582 (178) | 1646 (147) | 0.15 |
| tPA:Ag, ng/mL | 8.7 (3.9) | 8.1 (2.4) | 9.9 (4.6) | 0.33 |
| PAI-1:Ag, ng/ml | 13.9 (8.0) | 13.9 (7.1) | 12.4 (7.5) | 0.50 |

Data are shown as mean (standard deviation) or median (interquartile range). Abbreviations: see Supplemental Table 2.

Supplemental Table 6. Multinominal logistic regression model for central or peripheral recurrent PE episode in relation to recurrence-free patients.

| Variable | Central PE recurrence | | | | Peripheral PE recurrence | | | |
| --- | --- | --- | --- | --- | --- | --- | --- | --- |
|  | Univariate analysis | | Bivariate analysis | | Univariate analysis | | Bivariate analysis | |
|  | OR (95% CI) | p-value | OR (95% CI) | p-value | OR (95% CI) | p-value | OR (95% CI) | p-value |
| Age | 1.02 (0.97-1.06) | 0.48 |  |  |  |  |  |  |
| Male sex | 1.88 (0.63-5.59) | 0.26 |  |  |  |  |  |  |
| BMI | 0.99 (0.87-1.12) | 0.87 |  |  |  |  |  |  |
| Fibrinogen | 0.47 (0.22-0.99) | 0.047 |  |  |  |  |  |  |
| D-dimer | 1.005 (1.002-1.008) | 0.002 | 1.006 (1.003-1.09) | 0.001 | 1.003 (0.999-1.007) | 0.1 | 1.003 (0.999-1.007) | 0.11 |
| K_s_ | 0.92 (0.56-1.50) | 0.7 | 0.5 (0.36-1.18) | 0.16 | 0.54 (0.26-1.10) | 0.09 | 0.44 (0.20-1.02) | 0.06 |
| D-D_max_ | 0.84 (0.29-2.44) | 0.74 | 0.96 (0.34-2.74) | 0.95 | 0.84 (0.20-3.52) | 0.81 | 0.85 (0.20-3.63) | 0.83 |
| D-D_rate_ | 0.55 (0.26-1.18) | 0.12 | 0.46 (0.20-1.06) | 0.68 | 0.21 (0.06-0.74) | 0.02 | 0.19 (0.05-0.72) | 0.014 |
| peak thrombin | 1.003 (0.996-1.011) | 0.4 | 1.004(0.996-1.012) | 0.31 | 1.012 (1.004-1.020) | 0.003 | 1.013 (1.004-1.021) | 0.002 |
| ETP | 1.001 (0.995-1.007) | 0.8 | 1.001 (0.995-1.007) | 0.8 | 1.008 (0.999-1.017) | 0.07 | 1.008 (0.999-1.017) | 0.074 |
| tPA:Ag | 0.78 (0.62-0.98) | 0.03 | 0.79 (0.63-0.99) | 0.04 | 0.96 (0.74-1.25) | 0.78 | 0.96 (0.74-1.25) | 0.79 |

The final model was adjusted for fibrinogen level. BMI, body mass index; ETP, endogenous thrombin potential; D-D_max_, maximum D-dimer levels in the lysis assay; D-D_rate_, maximum rate of increase in D-dimer levels in the lysis assay; ETP, endogenous thrombin potential; K_s_, fibrin clot permeability coefficient; peak thrombin, peak thrombin concentration; tPA:Ag, tissue plasminogen activator antigen.
